# Explicit numerical solutions of a microbial survival model under nonisothermal conditions

**DOI:** 10.1002/fsn3.288

**Published:** 2015-11-14

**Authors:** Si Zhu, Guibing Chen

**Affiliations:** ^1^ Center for Excellence in Post‐Harvest TechnologiesNorth Carolina A&T State UniversityThe North Carolina Research Campus, 500 Laureate WayKannapolisNorth Carolina28081

**Keywords:** Explicit, Geeraerd model, microbial survival, nonisothermal, numerical solutions, parameter identification

## Abstract

Differential equations used to describe the original and modified Geeraerd models were, respectively, simplified into an explicit equation in which the integration of the specific inactivation rate with respect to time was numerically approximated using the Simpson's rule. The explicit numerical solutions were then used to simulate microbial survival curves and fit nonisothermal survival data for identifying model parameters in Microsoft Excel. The results showed that the explicit numerical solutions provided an easy way to accurately simulate microbial survival and estimate model parameters from nonisothermal survival data using the Geeraerd models.

## Introduction

Kinetic models for microbial survival during food pasteurization and sterilization processes are essential for design, assessment, optimization, and control of the processes. Microbial survival mostly exhibits nonlinear behavior (Van Boekel 2002; Heldman and Newsome 2003) which has been described by different mathematical models including the Weibull model (Peleg and Cole 1998), the biphasic model (Lee et al. 2001), the log–logistic model (Cole et al. 1993), the modified Gompertz (Linton et al. 1995), and the Geeraerd model (Geeraerd et al. 2000), among others. The selection of a suitable survival model is usually based on how well a model fits experimental survival data.

Parameters involved in a survival model depend on environmental conditions including presence of salt or acid, growth phase of the cells, the products or laboratory media used, and others because the heat resistance of a pathogen is influenced by these factors (Doyle et al. 2001). These parameters must be accurately estimated in order to use the models to evaluate the efficacy of a thermal process. Traditionally, they were estimated from a series of static survival curves. Because a true static condition is impossible to create and there may be more than one combination of parameters that give identical results (Dolan 2003), great efforts have been made to identify them by simultaneously fitting a survival model to each set of dynamic survival data using either software packages or self‐written computer programs (Peleg and Normand 2004; Valdramidis et al. 2008; Chen and Campanella 2012).

The Geeraerd model (Geeraerd et al. 2000) is frequently used to describe a type of non log‐linear microbial survival curves that show a shoulder and/or a tailing and it encompasses the first‐order inactivation when specific parameter values are selected. Under nonisothermal conditions, the model was formulated as a set of two coupled differential equations which could be solved using the Runge–Kutta method. The original Geeraerd model was also modified by incorporating a parameter expressed as a function of the heating rate to depict physiological adaptation induced by mild heat stress (Valdramidis et al. 2007).

The objectives of this study were to derive explicit numerical solutions of the original and modified Geeraerd models and to identify model parameters from nonisothermal microbial survival data using the numerical solutions in Microsoft Excel (Microsoft Corporation, Redmond, WA).

## Materials and Methods

### Microbial survival models

Under nonisothermal conditions, the Geeraerd model for microbial survival is expressed as the following equations (Geeraerd et al. 2000):

(1) $$\frac{dN(t)}{dt}=-k_{\max}\left(T\left(t\right)\right)\frac{1}{1+C_{c}\left(t\right)}\left(N\left(t\right)-N_{\mathrm{res}}\right)$$

(2) $$\frac{dC_{c}\left(t\right)}{dt}=-k_{\max}\left(T\left(t\right)\right)C_{c}\left(t\right)$$

where *N*(*t*) (CFU mL^−1^) represents the microbial cell density at time *t*,* C*_*c*_ (−) is related to the physiological state of cells, *N*_res_ (CFU mL^−1^) denotes the residual population density, and *k*_max_ (min^−1^) the specific inactivation rate which is temperature dependent. From equations (1) and (2), equation (3) was obtained (see Appendix S1).

(3) $$\log_{10}\frac{N(t)}{N(0)}=\log_{10}\frac{1+C_{c}\left(0\right)+\left(e^{\int_{0}^{t}k_{\max}\left(T\left(t\right)\right)dt}-1\right)\frac{N_{res}}{N(0)}}{C_{c}\left(0\right)+e^{\int_{0}^{t}k_{\max}\left(T\left(t\right)\right)dt}}$$

where *N*(0) (CFU mL^−1^) is the initial cell density and *N*(*t*)/*N*(0) survival ratio usually denoted by *S*(*t*), and *N*_res_/*N*(0) can be expressed as $10^{\log_{10}\frac{N_{\mathrm{res}}}{N(0)}}$ when *N*_res_ ≠ 0.

It was reported that exposure of *Escherichia coli* K12 to a mild thermal stress induced an increase in heat resistance and therefore a factor *k* was incorporated into equation (1) to account for this physiological adaption (Valdramidis et al. 2007).

(4) $$\frac{dN(t)}{dt}=-k_{\max}\left(T\left(t\right)\right)\frac{1}{1+C_{c}\left(t\right)}\left(N\left(t\right)-N_{\mathrm{res}}\right)k$$

where

(5) $$k=k_{1}\frac{dT/dt}{k_{2}+dT/dt}$$

where *k*_1_ and *k*_2_ are constants and *dT*/*dt* is the applied constant heating rate to raise temperature to a target value. From equations (2) and (4), equation (6) was obtained (see Appendix S2). When *k *=* *1, there is no detectable adaption of microbial cells to thermal stress and in this case equation (6) becomes equation (3).

(6) $$\begin{array}{c} \log_{10}\frac{N(t)}{N(0)}=\log_{10}\left\{{\left(\frac{1+C_{c}\left(0\right)}{C_{c}\left(0\right)+e^{\int_{0}^{t}k_{\max}\left(T\left(t\right)\right)dt}}\right)}^{k}\right.\\ \left.+\left[1-{\left(\frac{1+C_{c}\left(0\right)}{C_{c}\left(0\right)+e^{\int_{0}^{t}k_{\max}\left(T\left(t\right)\right)dt}}\right)}^{k}\right]\frac{N_{res}}{N(0)}\right\} \end{array}$$

Under nonisothermal conditions, a temperature profile can be represented by a series of discrete temperature points separated by sufficiently small time intervals Δ*t*. In this case, based on the Simpson's rule which approximates the value of a definite integral using quadratic polynomials, the integral term in equation (6) can be calculated by the following equation starting from $\int_{0}^{t_{1}=0}k_{\max}\left(T\left(t\right)\right)dt=0$

(7) $$\begin{array}{c} \int_{0}^{t_{n}}k_{\max}\left(T\left(t\right)\right)dt=\int_{0}^{t_{n-1}}k_{\max}\left(T\left(t\right)\right)dt+\frac{\Delta t}{6}\left[k_{\max}\left(T_{n-1}\right)+4k_{\max}\left(\frac{T_{n-1}+T_{n}}{2}\right)+k_{\max}\left(T_{n}\right)\right] \end{array}$$

where *n* is the number of temperature points (*n *≥* *2) and *T*_*n*_ the temperature value at time *t*_*n*_. Incorporating the values of the integral corresponding to a given discrete temperature profile into equation (3) results in the growth curve.

The specific inactivation rate *k*_max_ can be described by the Bigelow model (Bigelow 1921):

(8) $$k_{\max}=\frac{ln10}{AsymD_{\mathrm{ref}}}\exp\left[\frac{ln10}{z}\left(T-T_{\mathrm{ref}}\right)\right]$$

where *AsymD*_*ref*_ (min^−1^) denotes the asymptotic decimal reduction time at a reference temperature *T*_*ref*_ (°C) and *z* (°C) the temperature required for a 10‐fold change in *AsymD*_*ref*_ value. Parameters *AsymD*_*ref*_, *z*,* C*_*c*_ *(0),* and *log*_*10*_ *N*_*res*_ need to be determined. The value of *log*_*10*_ *N*(0) for each survival test can be experimentally measured at time zero or determined by curve fitting.

### Solving the differential equations using a MATLAB solver

The Geeraerd model (Geeraerd et al. 2000) under nonisothermal conditions was solved using the function ode45, a MATLAB's (MathWorks, Natick, MA) standard solver which uses a variable step Runge–Kutta method to solve differential equations numerically. The results obtained were compared with those calculated using equation (3) in Microsoft Excel under the same conditions.

### Microbial survival data

Equations (3), (7), and (8) and equations (5), (6), (7), (8) were fitted to nonisothermal survival data for *E. coli* K12 reported by Valdramidis et al. (2006, 2008), respectively, using the Microsoft Excel Solver. The data which were originally presented in plots were digitized by using the Digitizer Tool of Origin software (OriginLab Corporation, Northampton, MA) following the user guide.

### Parameter estimation using the Microsoft Excel solver

The procedure consists of the following four steps. A demonstration of the similar procedure was reported by Zhu and Chen (2015).

- Enter guesses for the model parameters to be identified in consecutive cells in Excel.
- Generate each survival table: For a survival test, enter discrete temperature profile in two adjacent columns of an Excel spreadsheet, time points being separated by intervals Δ*t* (min) (1/60 min for the present study). Then, calculate log_10_ *N*(*t*) or log_10_ *S*(*t*) at each time point by incorporating the guessed parameter values into equations (3), (7), and (8) or equations (5), (6), (7), (8).
- Look up calculated values of log_10_ *N*(*t*) or log_10_ *S*(*t*) for each survival test in its survival table: For a survival test, enter survival data in two adjacent columns of an Excel spreadsheet. For any data point log_10_ *N*(*t*) or log_10_ *S*(*t*), its calculated value is located in the log_10_ *N* column (assumed as *X* starting from row *Y*) of its growth table and row “=ROUND(*t*/Δ*t*,* 0*)+*Y*”. The formula: =ROUND (*t*/Δ*t*,* 0*) in Excel returns the nearest integer of *t*/Δ*t*. If the row number is contained in an assumed cell D#, then the formula: =INDIRECT(“*X*”&*D#*) returns the calculated log_10_ *N*(*t*) or log_10_ *S*(*t*).
- Minimize the overall sum of squared errors (SSE): Adding the SSE for each survival test yields the overall SSE, which is then minimized using the Excel Solver by changing the model parameter values. This optimization process results in the best‐fit of the model to the entire data sets.

The root mean squared error (RMSE) was used to evaluate the goodness of fitting of the model to microbial growth data using a reported formula (Neter et al. 1996).

## Results and Discussion

### Validation of the explicit numerical solutions

**Figure 1:**
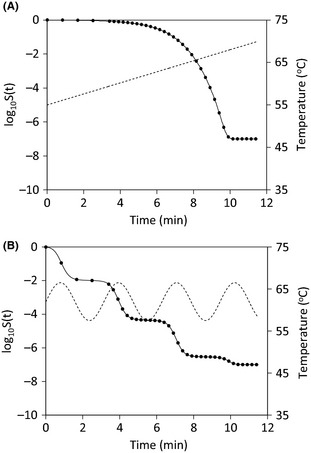
Survival curves calculated using equations (1), (2), and (8) (solid lines) and those using equations (3), (7), and (8) (filled symbols) under the same temperature conditions (dashed lines) (A) linear temperature profile, (B) sine temperature profile.

Microbial survival curves under two nonisothermal conditions were calculated by solving equations (1), (2), and (8) using the ode45 solver in MATLAB for given model parameters *AsymD*_*60*_ = 8 min, *z* = 5°C, log_10_(*N*_*res*_ /*N*(0)) = −7, and *C*_*c*_(0) = 1. Under the same conditions, survival curves were also calculated using equations (3) and (7) (Δ*t* = 1/60 min), and (8) in Excel. Survival curves obtained in these two methods are illustrated in Figure 1. Equation (3) was mathematically derived from equations (1) and (2). So they should be equivalent to each other. Such an equivalence was also visualized by Figure 1 which showed overlapped survival curves obtained in the two methods under the same conditions. Obviously, the explicit equation (3) provided a simpler method for the calculation. The accuracy of equation (3) depends on the accuracy of equation (7) which was used to numerically estimate $\int_{0}^{t}k_{\max}\left(T\left(t\right)\right)dt$. Theoretically, the error of the numerical approximation becomes negligible when time intervals are sufficiently small. Therefore, it is advised that temperature profiles are measured at small time intervals as possible. However, to reduce computation time, time intervals could be determined by gradually increasing it from a small value until the root mean squared difference between log_10_ *S*(*t*) in two consecutive calculations is smaller than a specified error tolerance.

### Identification of model parameters using the Microsoft Excel solver

**Figure 2:**
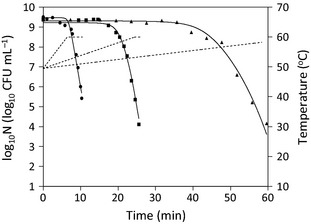
Published survival data for *Escherichia coli* K12 (filled symbols) (Valdramidis et al. 2008) under heat treatments (dashed lines) with a heating rate of 1.64°C min^−1^ (___), 0.43°C min^−1^ (___), and 0.15°C min^−1^ (___), respectively. Solid lines denote the fitted survival curves using equations (3), (7), and (8).

Figure 2 shows the fitting of equations (3) (*N*_*res*_ = 0), (7), and (8) to survival data for *E. coli* K12 (Valdramidis et al. 2008) under heat treatments with varying heating rates using the Microsoft Excel Solver. In the calculation, each temperature profile was converted to a series of discrete temperature points separated by constant time intervals of 1/60 min. The obtained model parameters and RMSE for curve fitting are illustrated in Table 1. As shown in the table, the value of RMSE (0.214 log_10_ CFU mL^−1^) obtained was relatively low, indicating the model fits the data well and this was also shown by the good agreement between the data points and the fitted curves in Figure 2. When an optimization procedure is used for curve fitting, model parameters must be assigned initial values which should be sufficiently close to their “true” values in order to make the optimization process converge to the “true” values (Chen and Campanella 2012). So, it is necessary to try different sets of guesses for the model parameters to find one that results in a desirably small RMSE for curve fitting. Results obtained from the same data by Valdramidis et al. (2008) were also included in Table 1. The table showed that the RMSE (1.16 log_10_ CFU mL^−1^) was four times greater than that obtained in the present study. So, the present study provided a more accurate curve fitting. The reason might be because the value of *C*_*c*_ *(0)* was not accurately identified in that report.

**Table 1:** Values of model parameters and RMSE obtained by fitting equations (3), (7), and (8) (*N*_*res*_ = 0) to published survival data of *Escherichia coli* K12 (Valdramidis et al. 2008) and those reported by Valdramidis et al. (2008)

Method	*AsymD*_54.75_	*z*	*C*_*c*_(0)	log_10_ *N*(0)_1_	log_10_ *N*(0)_2_	log_10_ *N*(0)_3_	RMSE
	(min)	(°C)		(log_10* *_CFU mL^−1^)	(log_10* *_CFU mL^−1^)	(log_10* *_CFU mL^−1^)	(log_10* *_CFU mL^−1^)
This study	10.35	4.97	70.13	9.48	9.23	9.32	0.214
Reported	10.05	5.02	1.92	9.41	9.23	9.30	1.16*

The RMSE was calculated using the given model parameters.

**Figure 3:**
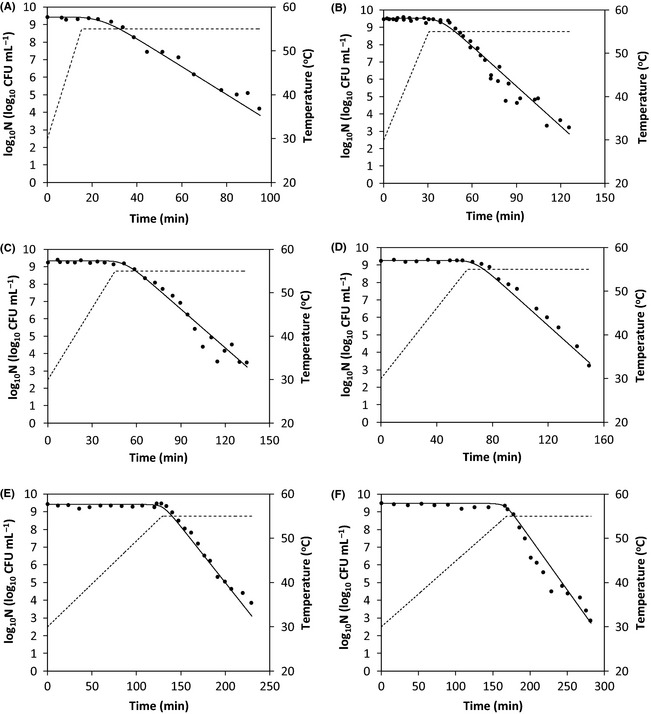
Published survival data for *Escherichia coli* K12 (filled symbols) (Valdramidis et al. 2006) and the fitted curves (solid lines) using equations (5), (6), (7), (8). Dashed lines denote temperature profiles with a heating rate of 1.64 (A), 0.82 (B), 0.55 (C), 0.40 (D), 0.20 (E), and 0.15 (F) °C min^−1^, respectively.

Thermal stress may increase heat resistance of microorganisms (Valdramidis et al. 2007; Corradini and Peleg 2009). To account for such effect, Valdramidis et al. (2007) proposed equations (4) and (5) to describe survival of *E. coli* K12 during heat treatment. Figure 3A–F illustrate nonisothermal survival curves of *E. coli* K12 measured at varying heating rates which delivered different extents of thermal stress to the microorganism (Valdramidis et al. 2006). Physiological parameters *k*_1_ and *k*_2_ in equation (5) were estimated by fitting equations (5), (6), (7), (8) (*N*_res_ *= *0) to the survival data. In the curve fitting, other model parameters including *AsymD*_ref_, *z*,* C*_*c*_ *(0)*, and *N(0)* were adapted from a previous report (Valdramidis et al. 2006) and fixed. The obtained values of *k*_1_, *k*_2_, and RMSE are listed in Table 2. The small RMSE indicated a good curve fitting. Because the microbial cells’ adaptation takes time, when the heating rate is sufficiently high*,* that is, *dT*/*dt *» *k*_2_, *k* should be equal to 1 which requires *k*_1_ = 1. This meant that *k*_1_ is constantly equal to 1 and thus is redundant in equation (5). As shown in Table 2, *k*_1_ obtained in this study was equal to 0.967 which agreed with the theoretical analysis.

**Table 2:** Values of *k*_1_, *k*_2_, and RMSE obtained by fitting equations (5), (6), (7), (8) to published survival data of *Escherichia coli* K12 (Valdramidis et al. 2006) and those reported by Valdramidis et al. (2007)

Method	*k*_1_ (−)	*k*_2_ (°C min^−1^)	RMSE (log_10_ CFU mL^−1^)
This study	0.969	0.060	0.368
Reported	0.696	0.042	0.787^a^

a The RMSE was calculated using the reported values of *k*_1_ and *k*_2_.

Parameters *k*_*1*_ and *k*_*2*_ were also identified from the same survival data by Valdramidis et al. (2007) using a two‐step method. The reported results are also listed in Table 2. Because this RMSE for curve fitting was one time greater than that in the present study, simultaneously fitting the survival model to all data sets resulted in more accurate parameter estimation.

The Geeraerd model (Geeraerd et al. 2000) essentially describes microbial survival curves that follow the traditional log‐linear model but have also a tailing and a shoulder. Explicit numerical solutions of both the original and the modified Geeraerd models provide a convenient and accurate way to identify model parameters and predict survival curves that follow the model under practical nonisothermal conditions.

## Conclusion

This study demonstrated that the two coupled differential equations used to describe the original or modified Geeraerd models could be simplified into an explicit equation. By numerically integrating the specific inactivation rate with respect to time involved in the equations, the obtained explicit numerical solutions could be conveniently used to accurately simulate microbial survival and estimate model parameters from nonisothermal survival data using only built‐in functions in Microsoft Excel. Because there is no need to solve differential equations, the explicit equations simplify the calculation and thus should facilitate practical applications of the Geeraerd models.

## Conflict of Interest

None declared.

## Supporting information


**Appendix S1.** Derivation of equation (3).**Appendix S2.** Derivation of equation (6).
