# Supplementary material for: Explicit numerical solutions of a microbial survival model under nonisothermal conditions
Source: Food Sci Nutr. 2015 Nov 14;4(2):284–9. doi: 10.1002/fsn3.288 (PMC4779493; doi:10.1002/fsn3.288)
Supplement: Supplementary file 1 — Appendix S1. Derivation of equation (3).Appendix S2. Derivation of equation (6). [file FSN3-4-284-s001.docx]

**Appendix S1**

**Derivation of Eq. (3)**

Eq. (9) can be obtained from Eq. (2) by integration.

$C_{c}(t)=\frac{C_{c}(0)}{e^{\int_{0}^{t} k_{\max}\left(T(t)\right)dt}}$ (9)

Substituting Eq. (9) for *C_c_*(*t*) in Eq. (1) and rewriting Eq. (1) yields (*N_res_* is a constant),

$\frac{d\left(N(t)-N_{res}\right)}{N(t)-N_{res}}= -\frac{k_{\max}\left(T(t)\right) e^{\int_{0}^{t} k_{\max}\left(T(t)\right)dt}}{C_{c}(0)+e^{\int_{0}^{t} k_{\max}\left(T(t)\right)dt}}dt$ (10)

Eq. (10) is equivalent to Eq. (11).

$d\ln\left(N(t)-N_{res}\right)=-d\ln\left(C_{c}(0)+e^{\int_{0}^{t} k_{\max}\left(T(t)\right)dt}\right)$ (11)

Integrating both sides of Eq. (11) with respect to *t* (starting at *t* = 0) gives,

$\ln\left(N(t)-N_{res}\right)| _{0}^{t}=-\ln\left(C_{c}(0)+e^{\int_{0}^{t} k_{\max}\left(T(t)\right)dt}\right) |_{0}^{t}$ (12)

Eq. (12) is equivalent to Eq. (13),

$\frac{N(t)-N_{res}}{N(0)-N_{res}}=\frac{1+C_{c}(0)}{C_{c}(0)+e^{\int_{0}^{t} k_{\max}\left(T(t)\right)dt}}$ (13)

Rearranging Eq. (13) yields,

$N(t)=\frac{N(0)\left(1+C_{c}(0)\right)+N_{res}\left(e^{\int_{0}^{t} k_{\max}(T(t))dt}-1\right)}{e^{\int_{0}^{t} k_{\max}(T(t))dt}+C_{c}(0)}$ (14)

Dividing *N*(0) on both sides of Eq. (14), and then taking log_10_ of both sides of the equation yields Eq. (3).

Under constant temperature conditions, rearranging Eq. (13) gives Eq. (14) which is the same as that given by Geeraerd *et al*. (2000).

$N(t)=\left(N(0)-N_{res}\right) e^{-k_{\max}t}\frac{1+C_{c}(0)}{1+C_{c}(0)e^{-k_{\max}t}}+N_{res}$ (15)

**Appendix S2**

**Derivation of Eq. (6)**

Substituting Eq. (9) for *C_c_*(*t*) in Eq. (4) and rewriting Eq. (4) yields,

$\frac{dN(t)}{N(t)-N_{res}}= -\frac{k\cdot k_{\max}e^{\int_{0}^{t} k_{\max}\left(T(t)\right)dt}}{C_{c}(0)+e^{\int_{0}^{t} k_{\max}\left(T(t)\right)dt}}dt$ (16)

Eq. (16) is equivalent to Eq. (17),

$d\ln\left(N(t)-N_{res}\right)=-k\cdot d\ln\left(C_{c}(0)+e^{\int_{0}^{t} k_{\max}\left(T(t)\right)dt}\right)$ (17)

Integrating both sides of Eq. (17)with respect to *t* (starting at *t* = 0) gives,

$\left. \ln\left( N\left( t \right)-N_{res} \right) \right|_{t=0}^{t}=-k\cdot\left. \left[ \ln\left( e^{\int_{0}^{t} k_{max}dt}+C_{c}\left( 0 \right) \right) \right] \right|_{t=0}^{t}$ (18)

Eq. (18) is equivalent to Eq. (19),

$\frac{N(t)-N_{res}}{N(0)-N_{res}}=\left(\frac{1+C_{c}(0)}{C_{c}(0)+e^{\int_{0}^{t} k_{\max}\left(T(t)\right)dt}}\right)^{k}$ (19)

Rearranging Eq. (19) yields,

$N\text{(}t)=N(0) \left(\frac{1+C_{c}(0)}{C_{c}(0)+e^{\int_{0}^{t} k_{\max}\left(T(t)\right)dt}}\right)^{k}+N_{res}\left[1-\left(\frac{1+C_{c}(0)}{C_{c}(0)+e^{\int_{0}^{t} k_{\max}\left(T(t)\right)dt}}\right)^{k}\right]$ (20)

Dividing *N*(0) on both sides of Eq. (20), and taking log_10_ of both sides of the equation yields Eq. (6).
